# Research on the Effect of Carbon Defects on the Hydrophilicity of Coal Pyrite Surface from the Insight of Quantum Chemistry

**DOI:** 10.3390/molecules24122285

**Published:** 2019-06-19

**Authors:** Peng Xi, Ruixin Ma, Wenli Liu

**Affiliations:** 1 Department of Environmental Engineering, North China Institute of Science and Technology, Beijing 101601, China; Maruixin@126.com; 2 School of Chemical and Environmental Engineering, China University of Mining and Technology, Beijing 10083, China; liuwenli08@163.com

**Keywords:** carbon defect, coal pyrite, surface, hydrophobicity, density functional theory

## Abstract

To investigate the effect of carbon defects on the hydrophilicity of the whole surface of the coal pyrite, the adsorption of the single H_2_O molecule at different sites of the coal pyrite surface was studied with the DFT calculation. It was found that, like the ideal pyrite, the single H_2_O molecule can stably adsorb at the doping-position, the ortho-position and the meta-position of the coal pyrite. The covalent bond and anti-bond were formed between O (water molecule) and Fe (the coal pyrite) through the Fe 3d orbital and O 2p orbital. Meanwhile, the S–H bond was replaced by the C–H bond. But away from the carbon defect centre, the adsorption of the single H_2_O molecule increased gradually and the Fe–O covalent bond strength between the single H_2_O molecule and the pyrite strengthened, which eventually became close to that of the undoped coal pyrite surface.

## 1. Introduction

Pyrite usually exists in magmatite rocks, contact metasomatic deposits and hydrothermal deposits. Coal pyrite is the pyrite produced in the coal environment and formed with the formation of coal. The floatability of pyrite is usually determined by its own nature, and the same is true for coal pyrite. The nature of coal pyrite is closely related to its lattice defects. Compared with pyrite, defects appear in the lattice of coal pyrite, and then the special physical and chemical properties form, which enhance its floatability [1]. To remove the coal pyrite during the flotation process efficiently, it is very important for the subsequent flotation desulfurization to study the hydrophobicity of the surface of coal pyrite and its forming mechanism. Many scholars have studied the relationship between the lattice defects of coal pyrite and its hydrophobicity [2,3,4]. The authors [5,6] found that there existed doped carbon on the coal pyrite surface and only simulated the effect of carbon defects on the doping-position of coal pyrite. This finding revealed the mechanism of change in hydrophobicity at the doping-position using DFT. However, the macroscopic hydrophobicity of coal pyrite not only reflects the hydrophobic change at the doping-position but also reflects the overall change in hydrophobicity at all regions of the coal pyrite surface. Meanwhile, it was not accurate to determine the structure and electronic properties of pyrite only using DFT, resulting in a small band gap of strongly associated semiconductor materials. Many authors often employed the DFT+U method to calculate the electronic structure and properties of the simulated pyrite and found that the band gap was closer to the experimental value of 0.95 eV [7]. Zhang et al. [8] explored the effect of different U values on the band gap and finally concluded that the bulk pyrite band gap was 1.02 eV with a U value of 2.0 eV. Sun et al. [9] came to a conclusion that when the U value was 2.0 eV, the band gap of the bulk pyrite was 1.03 eV. Additionally, Li et al. [10] found that the band gap was 0.95 eV using the GGA+U with a U value of 1.2 eV.

The Density Functional Theory (DFT) based on the first-principles method can give deep insights about the adsorption configuration between the adsorbates and the surface. Meanwhile, it can subsequently reveal the adsorption mechanism of the adsorbates on the surface. Chen et al. [11,12,13,14] and Li et al. [15,16] investigated the adsorption mechanism of H_2_O, CaOH, and O_2_ on the pyrite surface. The above study showed that it is feasible to calculate the adsorption of the molecule on the pyrite surface based on the Density Functional Theory. The previous study only [5,6] calculated the influence of the substituted and adsorbed carbon atoms on the adsorption of the single water molecule at the carbon atom doping- and adsorption position. The adsorption process, when the water molecule adsorbs on the other position of the whole one carbon atom-substituted pyrite surface, was not studied to reveal the mechanism of the whole coal pyrite’s hydrophilicity.

Based on the previous research, the adsorption processes of water molecules are simulated at the doping-position, ortho-position and meta-position of coal pyrite surface with the DFT+U method. The adsorption configuration, adsorption strength, bonding properties and bond strength, charge transfer and density of states of water molecules adsorbed at different positions on the coal pyrite surface containing carbon defects are compared with respect to adsorption energy, bond population and Mulliken charge population. The mechanism of the overall weakening of the hydrophilicity of coal pyrite under the condition of carbon defects is fully explained at the molecular level.

## 2. Calculation Methods and Model

### 2.1. Calculation Methods

In the process of structural optimizations, the module of CASTEP was used and the exchange-correlation interaction among electrons was described by the generalized-gradient approximation (GGA)-PW91. [17,18]. The interactions between the ionic cores and the valence electrons (Fe 3d^6^4s^2^, S 3s^2^3p4, C 2s^2^2p^2^) were modelled with ultra-soft pseudopotentials (USP) [19]. We used a cut-off of 350 eV for the plane-wave basis expansion [15] and a Monkhorst-Pack [20,21] k-point sampling density of 4 × 4 × 4 mesh. To more accurately describe the delocalization of electrons in the transition metal compound, the Hubbard U value of 1.2 eV was used to correct the orbit of Fe 3d. The Fe 3d was treated by the adoption of the Hubbard U correction in the paper and specified by tests in the previous paper [22,23]. 

Furthermore, we optimized the carbon atom and water molecule in a 20 × 20 × 20 Å cubic cell with Brillouin zone sampling restricted to the Gamma point in the calculation process. The other parameters were consistent with those reported above.

The strengths of the interactions between the adsorbates (water molecules) and the adsorbent (pyrite surface) were expressed by the adsorption energy (E_ads_) in the paper [24,25,26,27], defined as:

E_ads_ = E_X/slab_ − nE_X_ − E_slab_, (1)

where E_ads_ is the adsorption energy and X is the carbon atoms or water molecules. n is the number of carbon atoms or water molecules. E_X/slab_ is the energy of the pyrite surface with water molecules. E_slab_ and E_X_ are the energy of the pyrite surface and H_2_O, respectively. The more negative the E_ads_, the stronger the interaction between the adsorbate and mineral surface.

### 2.2. Surface Model

In the paper, the FeS_2_ (100) 2 × 2 × 1 supercell surface model cut from the optimized pyrite bulk cell was used as the undoped pyrite surface, which had 15 atomic layers, 15 Å vacuum layers and the bottom 9 atomic layers fixed. 

**Figure 1:**
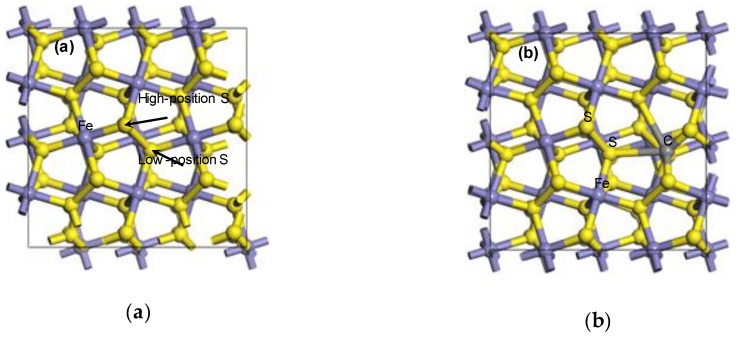

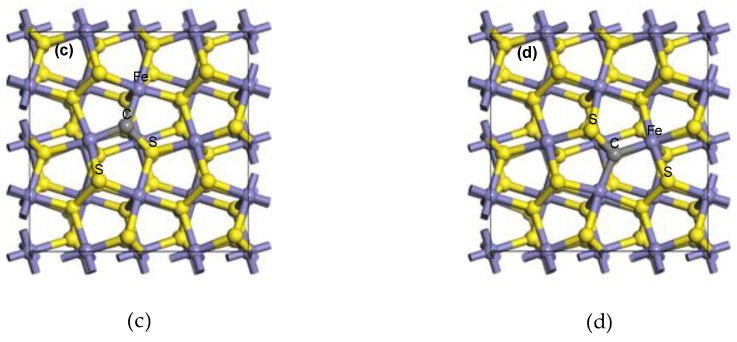
The doping model of one carbon atom-substituted pyrite surface: (**a**) I; (**b**) F; (**c**) HS; (**d**) LS.

The pyrite surface model doped with carbon atoms was optimized by impurity substitution energy [28]. The impurity substitution energy was the energy required to replace atoms in the surface lattice with impurity atoms, which measured the difficulty of substitution, defined as:

ΔE = E_total/C_ + E_X_ − E_total/perfect_ − E_C_, (2)

where △E is the impurity substitution energy and E_total/C_ is the energy of the pyrite surface substituted by the carbon atom. E_total/perfect_ is the energy of a perfect pyrite surface. E_X_ and E_C_ are the energies of the iron atom (or sulfur atom) and carbon atom, respectively. n is the number of carbon, sulfur or iron atoms. The △E was negative, which means that the substituted structure was feasible. The larger the negative value, the more stable the structure. The impurity substitution energy and substitution model are shown in Table 1 and Figure 1, respectively, after the sulfur and iron atoms of the ideal pyrite surface were substituted by carbon atoms.

It can be seen from Table 1 that △E was negative after the substitution of a carbon atom for a sulfur atom, and the △E of HS had a larger negative value. However, the △E of F was positive (△E = 51.2 kJ/mol). It was indicated that the sulfur atom of the pyrite surface could be spontaneously replaced by the carbon atom, and stable configurations were finally formed (shown in Figure 1c,d). Conversely, the iron atom can only be substituted by a certain amount of additional energy. The high-position sulfur atom and the low-position were, respectively, three coordination (unsaturated state) and four coordination (saturated state) so that it was easier for the high-position sulfur to be substituted. Simultaneously, because the properties of carbon atoms were close to those of sulfur atoms, but different from those of iron atoms, it was more difficult for iron atoms to be substituted, which was consistent with the theoretical calculations of isomorphism occurrence.

## 3. Results and Discussion

### 3.1. Adsorption Configurations and Adsorption Energies

**Figure 2:**
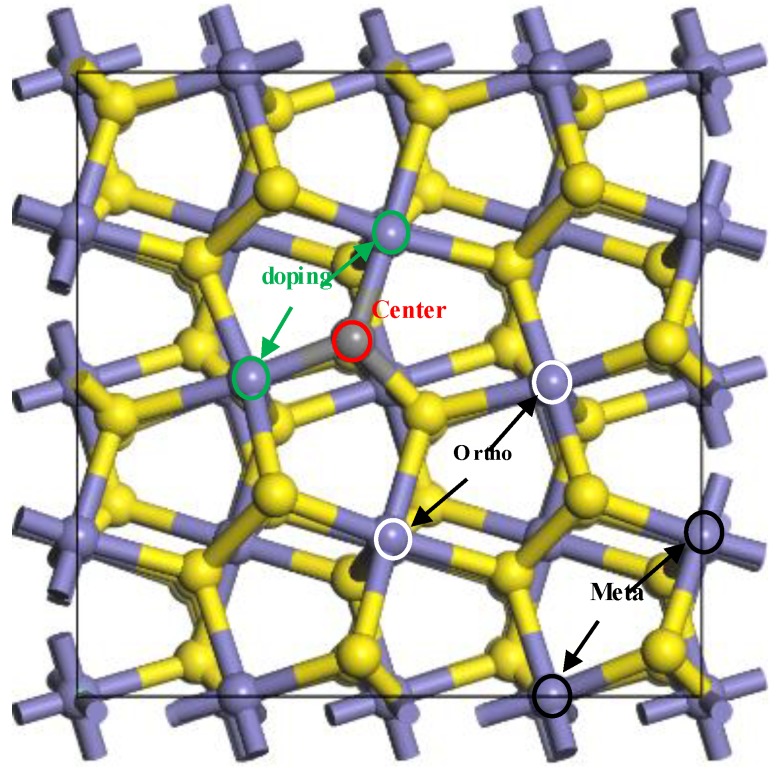
The different adsorption sites of H_2_O on the coal pyrite surface.

With the doped carbon atom as the centre of the coal pyrite, the “doping region” of coal pyrite means that the water molecule was adsorbed on the iron atom, which was connected to the carbon atom. The “ortho region” means that the adsorption site of the iron atom was not connected to the carbon atom and that the doped carbon atom did not directly participate in adsorption and bonding. The “meta region” means that the adsorption site was farther away from the centre, as shown in Figure 2. The “undoped region” means that the pyrite was ideal, with no carbon atom. The different adsorption sites of H_2_O on the coal pyrite surface are shown in Figure 2.

**Figure 3:**
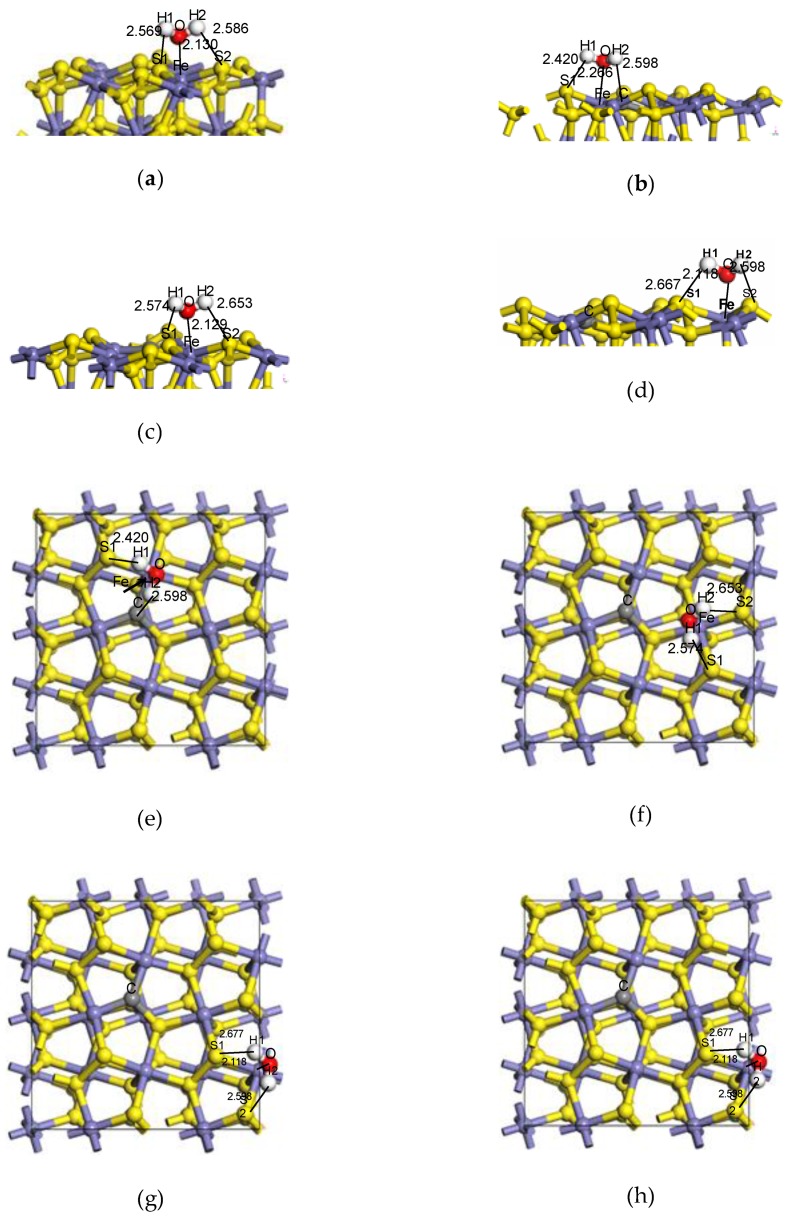
The adsorption configuration of H_2_O on different sites of the coal pyrite surface. Side view: (**a**) ideal surface; (**b**) doping-position; (**c**) ortho-position; Top view: (**d**) meta-position. (**e**) ideal surface; (**f**) doping-position; (**g**) ortho-position; (**h**) meta-position.

Previous studies [5,6] have shown that the adsorption model of H_2_O on the pyrite surface (in Figure 3a) was more stable, which was selected as the basic model to explore the influence of doped carbon atoms on the adsorption configurations, E_ads_, bonding, charge transfer and density of states (DOS) of H_2_O on the pyrite surface in the paper. 

The adsorption configurations and adsorption energies of water molecules on the ideal pyrite surface and the doping-position, ortho-position and meta-position of the surface doped with carbon atoms are shown in Figure 3 and Table 2. 

Our results revealed that the adsorption energies of the water molecule at the doping-position, ortho-position and meta-position of the pyrite surface doped with carbon atoms were lager than that of the ideal surface, and the adsorption energy respectively decreased, which gradually approached but remained lower than the E_ads_ (−79.19 kJ/mol) of the ideal surface [22]. These results suggest that the water was easier to adsorb on the ideal surface and it was indicated that H_2_O can still be spontaneously adsorbed on any position of the pyrite surface doped with carbon atoms, but only the adsorption strength was relatively weaker. Meanwhile, with the distance from the doping centre, the negative value of adsorption energy of H_2_O on the surface increased gradually. The doping carbon atom had the greatest influence on the adsorption of H_2_O at the doping site, and the farther away from the doping centre, the smaller the adsorption effect. 

The above results showed that the macroscopic overall weakening of the hydrophilicity of coal pyrite doped by carbon atoms was due to the weakening of the adsorption of the water molecule at various positions (doping-position, ortho-position and meta-position) on the surface, which ultimately resulted in the coal pyrite showing the property of easier flotation during the process of slime flotation. 

### 3.2. Analysis of Bonding

The Mulliken population [29] showed the strength of the interaction between the bonding atoms after the adsorbate adsorbed on the adsorbent. Generally, the larger the bond Mulliken population, the shorter the bond length, and the stronger the covalent bond formed between atoms. The bond Mulliken population and bond length after adsorption of H_2_O on different sites of the pyrite surface are shown in Table 3. 

After H_2_O was adsorbed on the modified pyrite surface, the Fe–O covalent bond was formed and the bond population increased and approached that of the ideal surface gradually [23]. The Mulliken population results showed that the strength of the Fe–O bond increased gradually, and the farther away from the doping centre of carbon atoms, the more stable the adsorption of the water molecule was, which was in accordance with the results of E_ads_.

**Figure 4:**
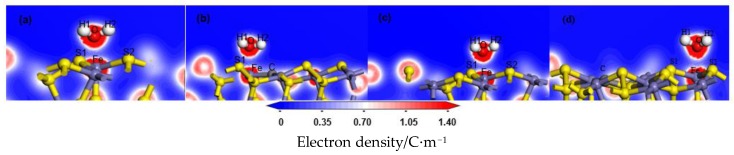
Electron density after H_2_O adsorption on the coal pyrite surfaces: (**a**) ideal surface; (**b**) doping-position; **(c**) ortho-position; (**d**) meta-position.

**Figure 5:**
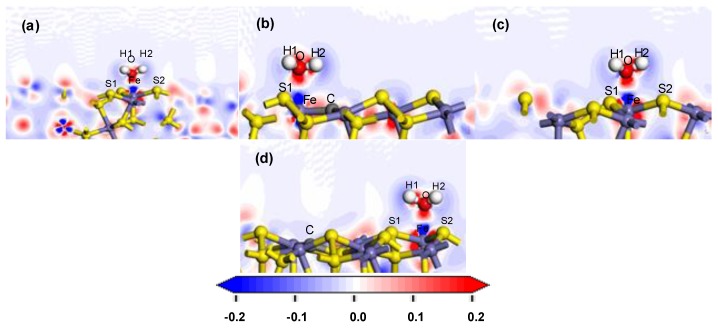
Charge density difference after H_2_O adsorption on the coal pyrite surface: (**a**) ideal surface; (**b**) doping-position; (**c**) ortho-position; (**d**) meta-position.

The electron density and the charge density difference with respect to both O and Fe were further analysed. The deeper the red in the charge density map, the greater the charge density there. The red was deeper and therefore exhibited more electron cloud between O and Fe, which indicated that the bond between them was strong, as shown in Figure 4. In the charge density difference map, the red region indicated an increase in electron density, and the blue region indicated a decrease, as shown in Figure 5. With the deviation from the doping centre, there were more charge density distributions and obvious charge transfers between the iron atom and the oxygen atom, which gradually approached but remained lower than that of the ideal surface. These results indicated that the interaction between the water molecule and the modified pyrite surface became stronger gradually with the distance of the doping centre, but was lower than that of the ideal surface.

### 3.3. The Charge Transfer

The charge transfer refers to the loss and transfer of electrons between atoms after adsorption of adsorbate on the surface of the adsorbent, which was expressed by the Mulliken charge population (MCP), as shown in Table 4, Table 5, Table 6 and Table 7, before adsorption (BA) and after adsorption (AA) of H_2_O on the surface of the ideal pyrite and at different positions of pyrite doped with carbon atoms. 

Similar to the MCP results of atoms on the ideal surface, after the adsorption of H_2_O at the different sites of the pyrite surface with carbon defects, whether the water molecule adsorbed at the doping-position, ortho-position or meta-position, the negative charge of the oxygen atom in the water molecule also decreased due to the loss of approximately 0.20 e of the O 2p orbital, and the positive charge of the Fe atom on the surface also increased due to the gain of approximately 0.07 e, which may be the similar adsorption results of the single water molecule at various positions. Finally, electron aggregation enabled H_2_O to be stably adsorbed on the pyrite surface.

### 3.4. The Density of States (DOS)

The interaction between H_2_O and the coal pyrite was mainly through the interaction between O of the former, and C and Fe of the latter. Therefore, only the PDOS values of O, C and Fe were plotted before and after adsorption [30].

**Figure 6:**
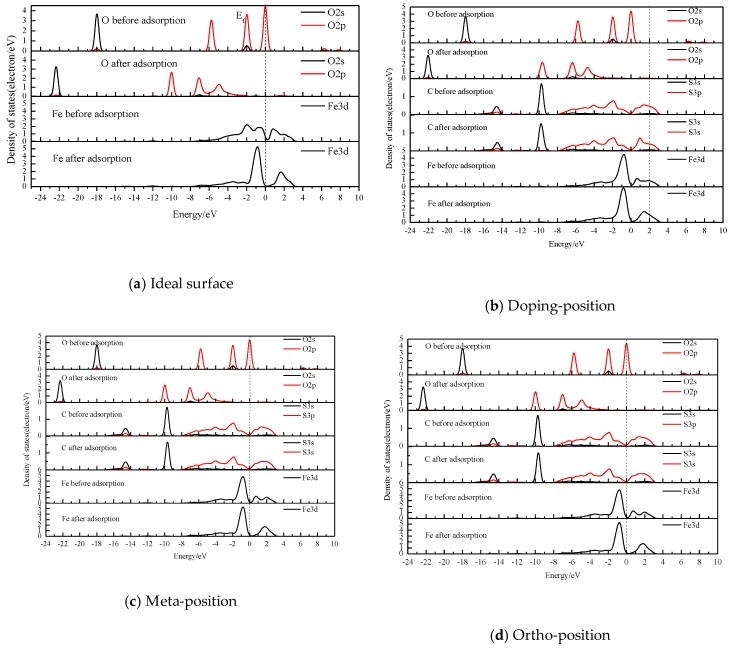
Density of states (DOS) of atoms before and after H_2_O adsorption at different sites of pyrite surfaces.

After H_2_O was adsorbed on the different locations of the pyrite surface doped by carbon atoms (shown in Figure 6b–d), similarly to the results of the ideal surface [19], the covalent bond and anti-bond were formed between the water molecule and the pyrite surface through the O 2p orbital and the Fe 3d orbital. The bonding energy between Fe and O was very strong, while the anti-bonding was quite weak. However, the strength of the covalent bond increased with the distance from the carbon defect centre, which was close to that of the ideal pyrite surface. 

## 4. Conclusions

(1) The calculation results of the adsorption energy showed that whether the water molecules were adsorbed on the doping-position, the ortho-position or the meta-position of the coal pyrite, the water molecules can adsorb on all positions of the surface spontaneously. With the distance from the defect centre, the negative value of adsorption energy increased gradually, which was close to that of the ideal surface. 

(2) The Fe–O covalent bond was mainly formed after adsorption, and the strength of covalent bonding was doping-position < ortho-position < meta-position, lower than the ideal surface. The interaction between the water molecule and the pyrite surface mainly depended on the covalent bonding and anti-bonding through the 2p orbital of the oxygen atom and the 3d orbital of the iron atom. 

(3) It was not only shown that, compared with the undoped surface, the hydrophilicity of the coal pyrite surface with carbon defects decreased at the defect site, but also that the degree of hydrophilicity reduction decreased gradually with the distance of the ortho-position and the meta-position from the defect centre. Whatever the doped site or the undoped site, the hydrophilicity decreased. The weakening of the hydrophilicity at the doping-position, ortho-position and meta-position resulted in the overall weakening of the hydrophilicity on the coal pyrite surface from the molecular level.

## Figures and Tables

**Table 1:** Impurity substitution energy of the pyrite surface substituted by carbon atoms on different sites.

Substitution Site	△E/kJ/mol
F	51.24
HS	−126.90
LS	−58.12

Note: For the sake of simplicity, I represents the model of the ideal pyrite surface. F represents the surface model after replacing iron atoms with carbon atoms. HS represents the surface model after replacing the high-position S atoms with carbon atoms. LS represents the surface model after replacing the low-position S atoms with carbon atoms.

**Table 2:** E_ads_ of H_2_O on different sites of the pyrite surface with carbon defects.

Adsorption Configuration	E_ads_/(kJ/mol)
doping-position	−50.18
ortho-position	−67.13
meta-position	−69.35

**Table 3:** Mulliken population after H_2_O adsorption on different sites of the coal pyrite surface.

Adsorption Model	Bond	Population	Length
doping-position	Fe–O	0.10	2.266
	H1–S1	0.01	2.420
	H2–C	−0.00	2.598
ortho-position	Fe–O	0.13	2.129
	H1–S1	0.01	2.574
	H2–S2	−0.01	2.653
meta-position	Fe–O	0.13	2.118
	H1-S1	−0.01	2.667
	H–-S2	0.01	2.598

**Table 4:** Mulliken charge population (MCP) of atoms on the ideal pyrite surface.

Atomic Label	Adsorption Status	s	p	d	T	Charge/e
Fe	BA	0.40	0.52	6.90	7.82	0.18
	AA	0.32	0.42	7.10	7.84	0.16
O	BA	1.89	5.16	0.00	7.05	−1.05
	AA	1.87	4.98	0.00	6.84	−0.84
H1	BA	0.47	0.00	0.00	0.47	0.53
	AA	0.54	0.00	0.00	0.54	0.46
H2	BA	0.47	0.00	0.00	0.47	0.53
	AA	0.53	0.00	0.00	0.53	0.47
S1	BA	1.86	4.27	0.00	6.14	−0.14
	AA	1.85	4.30	0.00	6.15	−0.15
S2	BA	1.87	4.29	0.00	4.16	−0.16
	AA	1.85	4.32	0.000	6.17	−0.17

**Table 5:** MCP of atoms at the doping-position.

Atomic Label	Adsorption Status	s	p	d	T	Charge/e
Fe	BA	0.33	0.37	7.08	7.78	0.22
	AA	0.31	0.38	7.03	7.72	0.28
O	BA	1.89	5.16	0.00	7.05	−1.05
	AA	1.87	4.97	0.00	6.85	−0.85
H1	BA	0.47	0.00	0.00	0.47	0.53
	AA	0.54	0.00	0.00	0.54	0.46
H2	BA	0.47	0.00	0.00	0.47	0.53
	AA	0.53	0.00	0.00	0.53	0.47
S1	BA	1.86	4.28	0.00	6.14	−0.14
	AA	1.85	4.32	0.00	6.17	−0.17
C	BA	1.60	2.90	0.00	4.50	−0.50
	AA	1.60	2.91	0.00	4.51	−0.51

**Table 6:** MCP of atoms at the ortho-position.

Atomic Label	Adsorption Status	s	p	d	T	Charge/e
Fe	BA	0.34	0.43	7.15	7.92	0.08
	AA	0.32	0.42	7.10	7.84	0.16
O	BA	1.89	5.16	0.00	7.05	−1.05
	AA	1.86	4.98	0.00	6.85	−0.85
H1	BA	0.47	0.00	0.00	0.47	0.53
	AA	0.54	0.00	.0.00	0.54	0.46
H2	BA	0.47	0.00	0.00	0.47	0.53
	AA	0.53	0.00	0.00	0.53	0.47
S1	BA	1.86	4.25	0.00	6.10	−0.10
	AA	1.85	4.30	0.00	6.15	−0.15
S2	BA	1.86	4.27	0.00	6.13	−0.13
	AA	1.85	4.30	0.00	6.15	−0.15

**Table 7:** MCP of atoms at the meta-position.

Atomic Label	Adsorption Status	s	p	d	T	Charge/e
Fe	BA	0.34	0.43	7.14	7.91	0.09
	AA	0.32	0.42	7.10	7.84	0.16
O	BA	1.89	5.16	0.00	7.05	−1.05
	AA	1.86	4.98	0.00	6.84	−0.84
H1	BA	0.47	0.00	0.00	0.47	0.53
	AA	0.53	0.00	0.00	0.53	0.47
H2	BA	0.47	0.00	0.00	0.47	0.53
	AA	0.54.	0.00	0.00	0.54	0.46
C	BA	1.60	2.90	0.00	4.50	−0.50
	AA	1.60	2.90	0.00	4.50	−0.50
